# Seroprevalence Survey of American Trypanosomiasis in Central Valley of Toluca

**DOI:** 10.1100/2012/450619

**Published:** 2012-04-30

**Authors:** Israel A. Quijano-Hernández, Alejandro Castro-Barcena, Alberto Barbabosa-Pliego, Laucel Ochoa-GarcÍa, Javier Del Ángel-Caraza, Juan C. Vázquez-Chagoyán

**Affiliations:** ^1^Hospital Veterinario para Pequeñas Especies, Facultad de Medicina Veterinaria y Zootecnia, Universidad Autónoma del Estado de México, C.P. 50130 Toluca, MEX, Mexico; ^2^Centro de Investigación y Estudios Avanzados en Salud Animal, Facultad de Medicina Veterinaria y Zootecnia, Universidad Autónoma del Estado de México, C.P. 52000, Toluca, MEX, Mexico

## Abstract

American trypanosomiasis is a growing health issue in the Americas. México is an endemic country, where some locations such as in the State of México are considered highly prevalent. In the valley of Toluca city, the capital of the State of Mexico, there exists an apparent high prevalence in dogs. The absence of triatomine vectors suggests that dogs may not be infected. Therefore, we conducted a directed survey to domiciliated and nondomiciliated dogs to reassess dogs' *T. cruzi* seroprevalence status. HAI and ELISA serologic tests were applied to 124 and 167 serums of domiciliated and nondomiciliated dogs in the target city. Risk factors were estimated, but the results did not show any evidence to assess them. No domiciliated dogs tested positive to both tests, whereas only one non-domiciliated dog resulted positive. This animal may have acquired the infection in an endemic area and then migrated to Toluca. Research results indicate that *T. cruzi* infection is not actively transmitted among dogs, and it is pointed out that dogs are the main sentinel animal population to evaluate a possible expansion of the territory affected by Chagas' disease.

## 1. Introduction

Chagas disease is known as one of the most important forgotten health problems in the Americas. *Trypanosoma cruzi* (*T. cruzi*) is defined as a hemoflagellate parasite responsible for long-term cardiac and gastrointestinal pathologies in human beings. *T. cruzi *may be transmitted by many species of triatomines like the “kissing bug.” *Triatoma dimidiata, Rhodnius prolixus,* and *Triatoma infestans* are considered among the most important vectors in America [1]. In Mexico, more than 30 vectors of* T. cruzi* species have been described, and some of the most important species considered in our country are *T. dimidiata*,* T. pallidipennis*,* and T. barberi* [2].

Mexico is considered an endemic country, where the* T. cruzi* is transmitted through vectors as described in previous studies. Most of the states in Mexico have been shown positive seroprevalences among humans [2], as it has been observed in other countries. The highest risk areas in Mexico are those rural communities located under 2000 meters according to the sea level, where human communities have invaded triatomines natural habitats and even have used materials that allow the colonization of these arthropods [3].

The State of Mexico was considered free of the disease until the early 2000s, when health authorities found few cases of Chagas' disease among humans. Later, Estrada-Franco et al., [4] confirmed the existence of the disease in the State of Mexico reporting the first epidemiologic study. In that study, and in more recent ones, [5] demonstrated that, in the southern communities of the State of Mexico, dogs' seroprevalence is nearly 25%, and 34% of triatomine specimens were infected with *T. cruzi*. Furthermore *T. cruzi* isolated from *Triatoma pallidipennis* obtained from a little community called Zumpahuacan, located at the southern of the State of Mexico, was highly pathogenic for dogs. 

Different domestic animals are involved in the domiciliary and peridomiciliary cycles of* T. cruzi*. Dogs and cats are considered epidemiologically important, due to the fact that they are considered reservoirs; they are a source of infection for bugs, which may reach and infect humans. It has been shown that dogs represent a highly risk factor for human infection than cats, [6]. In addition to this, the presence of dogs increases up to 9 times the probability of humans to be infected with *T. cruzi* in highly endemic areas [7]. As a consequence, dogs have been considered an excellent marker or sentinel to evaluate the presence of *T. cruzi* infections and the evolution of treatments [8, 9] and are epidemiologically relevant for Chagas disease studies as some reports, where seroprevalences to *T. cruzi* were found in putatively nonendemic areas have described [4, 10]. Thus, we considered it relevant, before proposing a measure that could cause distress in pet owners, to conduct another epidemiologic study in dogs from Toluca Valley in order to accept or reject previous results. As a result, we studied *T. cruzi* seroprevalence of domiciliated and nondomiciliated dogs, to reassess dogs' Chagas disease status in Toluca Valley.

## 2. Materials and Methods

### 2.1. Study Area

The study was conducted in Toluca Valley, the Sate of Mexico capital, (19°37′32′′N latitude and 99°39′14′′W longitude) located at an altitude of 2640 meters over the sea level. Under the Koppen climate classification, Toluca features a subtropical highland climate with a rainy season during the summer from June to September (INEGI 1999).

### 2.2. Dogs

Nondomiciliated dogs (*n* = 167) in a municipal canine control center and domiciliated dogs (*n* = 124) assisted in a veterinary teaching hospital were serologically tested to detect IgG anti-*T. cruzi* antibodies. ELISA and HAI tests were applied using 3 mL. venous blood samples taken from cephalic or jugular vein (depending on the dog size). Domiciliated dogs were grouped according to their similar backgrounds (origin, age, breed, and sex) and some owners' information dealing with the dog's assistance at chagasic endemic areas.

Blood samples were centrifuged at 1800 g during 10 min at a room temperature. Dogs' serum (supernatant) was collected and stored at −20°C previously to the analysis. 

Detection of anti-*T. cruzi *antibodies was conducted using two serologic kits: indirect hemagglutination assay (IHA) (Polychaco, Laboratorio-Lemos SRL, Buenos Aires, Argentina) with 98% of sensitivity and 99% of specificity, according to the manufacturer's specifications and enzyme-lynked immunosorbent assay (ELISA) (Laboratorio-Lemos SRL, Buenos Aires, Argentina) with 100% of sensitivity and 100% of specificity, according to the manufacturer's specifications. Assays were used cautiously except that horseradish peroxidase (HRP) labeled anti-human-IgG antibody in the ELISA kit was replaced with horseradish peroxidase-labeled anti-dog-IgG (Koma Biotech, Seoul, Korea). The cut-off value for IHA (positive titer at ≥1 : 16 serum dilution) and ELISA (0.129 OD_450 nm_  + 2 SD) were established using the serum from healthy dogs (*n* = 30, 1 : 100 dilution) as previously described, dogs were considered positive when reactive for both assays [5].

Univariate analysis (*χ* ^2^) to define associations of possible risk factors and seropositivity of specific antibodies against *T. cruzi *were applied. Odds ratio (OR) and 95% CI were included. The level of significance was established at *P* < 0.05.

## 3. Results

24 male and female domiciliated dogs are different pure and nonpure breeds. Ages are ranging from 3-month to 16-year old. Dog's nativeness was defined considering the time they had lived in the area, that is, since they were two months old to the current day of the study. A small rate was confirmed to have visited endemic areas previously. Owners showed familiarization with the “kissing bug” name, but it was not possible for them to identify it. From 3 months to 14-year old, nondomiciliated dogs were considered nonpure breed, according to the veterinarian's description base on the dental formula and tooth conditions.

Domiciliated dogs resulted all negative to both tests (ELISA and IHA), and only one male nondomiciliated dog (approximately 3-year old) was reactive to the two referred tests and considered positive (Table 1), and risk factor analysis was not further considered.

## 4. Discussion

The study investigated whether dogs in Toluca Valley are a source to infect *T. cruzi* to humans. Outcomes show that apparently high prevalence previously reported may be associated to a specific group of dogs that were probably under constant mobilization to chagasic endemic areas [4, 10]. This study clearly confirms that Chagas is not an endemic disease in domiciliated and nondomiciliated dogs in Toluca Valley. Thus, Toluca's dogs do not represent a significant risk factor for *T. cruzi* human infection. Moreover, this negativity assumption may be considered to evaluate a possible dissemination of *T. cruzi* beyond its current geographical restriction considering the climatic change, due to the fact that dogs are important sentinels for the disease [8, 11]. Results suggest that the absence of triatomine vectors is a meaningful factor that explains the low prevalence detected. The reason is that vectors are the main source for canine infection [9]. Although* T. cruzi* transmission from dog to human without the intermediary vector has not been reported, the eventual risk of accidental human infection through dogs' blood could exist, and preventive measures should be taken in Toluca's valley dogs.

The risk of natural infection is negligible in Toluca Valley. Human infection may derive from infected people migrating from endemic areas, from blood transfusion or transplanted organs from infected donors, rather than from vectorial transmission. The presence of seropositive dogs in houses, where vectors are well adapted, put people at risk to be infected through the bite of an infected vector of a positive dog [12]. Result should be considered cautiously due to the varied insects species extending to other geographical areas with higher altitudes and latitudes, where weather conditions are more suitable and less harsh for them as a consequence of the global warming. Butterflies, beetles, dragonflies, and grasshoppers illustrated this geographical adaptation mentioned above [13]. Triatomines are not as mobile as other insects. Consequently, natural migration could no be a fast process. Yet constant human migration between endemic to nonendemic areas that have become warm enough for the target insects may accelerate the insects' expansion to the new geographical areas.

The study reports and evaluates seroprevalence design for dogs' population in Toluca valley city. Findings show that *T. cruzi *infection is not actively transmitted among dogs. It is pointed out that dogs are the main species to assess the likely mobilization of Chagas' disease due to climatic change.

## Figures and Tables

**Table 1:** Serology test results from dogs.

	HAI		ELISA		
Dog/Result	Positive	Negative	Positive	Negative	Total
Domiciliated	0	124	0	124	124
Nondomiciliated	1	166	1	166	167

Total	1	290	1	290	291

Seropositivity was estimated in 0.34%, apparent prevalence 0.34%, and true prevalence of 0.24%.
